# Mental Representations of Illness in Patients with Gestational Trophoblastic Disease: How Do Patients Perceive Their Condition?

**DOI:** 10.1371/journal.pone.0153869

**Published:** 2016-04-21

**Authors:** Valentina E. Di Mattei, Letizia Carnelli, Martina Mazzetti, Martina Bernardi, Rossella Di Pierro, Alice Bergamini, Giorgia Mangili, Massimo Candiani, Lucio Sarno

**Affiliations:** 1 Vita-Salute San Raffaele University, Milan, Italy; 2 Clinical and Health Psychology Unit, IRCCS San Raffaele Hospital, Milan, Italy; 3 Department of Psychology, University of Milano-Bicocca, Milan, Italy; 4 Obstetrics and Gynecology Unit, IRCCS San Raffaele Hospital, Milan, Italy; University of Missouri, UNITED STATES

## Abstract

**Background:**

Gestational Trophoblastic Disease comprises a group of benign and malignant disorders that derive from the placenta. Using Leventhal’s Common-Sense Model as a theoretical framework, this paper examines illness perception in women who have been diagnosed with this disease.

**Methods:**

Thirty-one women diagnosed with Gestational Trophoblastic Disease in a hospital in Italy were asked to complete the Illness Perception Questionnaire-Revised to measure the following: illness Identity, illness opinions and causes of Gestational Trophoblastic Disease.

**Results:**

High mean scores were observed in the Emotional representations and Treatment control subscales. A significant difference emerged between hydatidiform mole patients and those with gestational trophoblastic neoplasia on the Identity subscale. A significant correlation emerged between “time since diagnosis” and the Treatment control subscale.

**Discussion:**

This study is the first to investigate illness perception in Gestational Trophoblastic Disease. From a clinical perspective the results highlight the need for multidisciplinary support programs to promote a more realistic illness perception.

## Introduction

Gestational trophoblastic disease (GTD) encompasses a group of disorders that derive from the placenta, including the premalignant complete and partial hydatidiform mole (HM), the malignant invasive mole (IM), choriocarcinoma (CCA), placental site trophoblastic tumor (PSTT) and epithelioid trophoblastic tumor (ETT) [1]. The malignant conditions are known as gestational trophoblastic neoplasia (GTN).

The incidence of hydatidiform mole is varied, ranging from 1.5 to 6 per 1000 pregnancies in North America and Europe; in Italy the estimated frequency of HM over the 1996–2008 time period was 1 case every 935 pregnancies [2, 3]. Data for GTN are more limited because the diagnosis is generally made clinically rather than on biopsy material. In Europe and North America choriocarcinoma affects approximately 1 in 40,000 pregnancies, while for PSTT the incidence is estimated at 0.2% of all GTD [1].

Although previously a lethal disease, GTD is presently considered one of the most curable gynecological cancers. This progress can be attributed to several factors including the high tumor chemosensitivity, the incorporation of aggressive multimodality therapy, the presence of a tumor marker; the beta subunit of human chorionic gonadotropin (β-hCG). Appropriate chemotherapy and surgery result in excellent survival rates (approaching 100%) [1] with fertility being maintained in the majority (80%) of women with post-molar GTN [1].

Treatment depends on the individual patient, the type of tumor and the absence/presence of metastases. It may include surgery (suction evacuation and curettage or hysterectomy) and chemotherapy (e.g. with Methotrexate) [1] which is indicated when a plateau or a rise in β-hCG occurs, and in presence of GTN [4]. Follow-up consists in weekly β-hCG level monitoring until undetectable (<5 mIU/ml) for 3 weeks, then monthly monitoring for at least 6 months [1]. After chemotherapy, the follow-up goes on for at least 1 year [4]. During this period women are advised not to get pregnant and to practice contraception at least until the end of follow-up [5]; the resulting β-hCG production in pregnancy can in fact hinder detection of post-molar progression to GTN [1, 6].

GTD diagnosis, treatment and follow-up present a sudden and prolonged factor of stress, which forces the patient and her partner to find a new psychological accommodation [7]. Fear of the disease, waiting for normalization of β-hCG during follow-up, concerns about fertility and worries regarding future pregnancies are the main determinants of anxiety and distress among GTD patients [8–11].

Several theories and models have highlighted how patients’ beliefs and perceptions of their disease and symptoms are important factors for psychological adjustment to their condition [12, 13]. The most influential theoretical framework adopted in this area of study is the Common-Sense Model of Illness Representation (CSM) proposed by Leventhal and colleagues [14]. The CSM hypothesizes that individuals create mental representations of their illness on the basis of the concrete and abstract sources of information available to them, in order to make sense of and manage the problem [13]. According to this theory, a response to a health threat is the result of an underlying control system, which can be divided into three broad processes. The first is the construction of a representation of the health threat. This representation is assumed to be based on three sources of information: the general pool of “lay” information that an individual has previously assimilated, the knowledge derived from friends, family and authoritative sources and the current experience of the illness (such as somatic experiences and symptoms) [15]. The second stage involves the development of an action plan in which individuals use the coping strategies they perceive and believe to be appropriate. The third phase is the process of coping appraisal, which consists in the evaluation of the coping strategies’ effectiveness on the outcome or goal. The CSM assumes that these three stages occur in parallel on both an emotional and cognitive level, and it underscores that the interaction between each level is dynamic, so that each component is influenced by a process of feedback [16]. Many studies in this field, based on different methodologies across a range of clinical conditions, have established that the content of an illness representation can be ordered into the following logical themes or dimensions [17–19]:

1. Identity: the label given to the condition and the symptoms the patient perceives to be related to his or her illness;
2. Cause: the beliefs regarding the factors that are responsible for causing the illness or disease, which may not be completely biomedically accurate. These may include single causes or more complex multiple causal models;
3. Timeline: the individual’s belief about the course of the illness and time scale of illness symptoms. These beliefs will be re-evaluated as time progresses. They can be categorized into acute, chronic or episodic;
4. Consequences: the individual beliefs about the outcomes of the condition and how this will impact on overall quality of life or how it may affect one’s functional capacity;

Recent research has resulted in the inclusion of another illness representation dimension: the belief about the *cure* or *controllability* of an illness [20]. This dimension refers to the sensation of empowerment regarding performance of coping behavior or the efficacy of a treatment.

The CSM has been used with success to explain psychological and physical outcomes in a range of acute and chronic illnesses [13]. There is also evidence that the model is useful in predicting psychological well-being in different clinical conditions [21, 22]. Moreover, these representations operate at multiple levels and could guide patients’ preferences for treatment and the behaviors in which they engage over time.

To our knowledge, Illness Perception has never been analyzed in GTD patients. Using the literature as a starting point, we designed a study with the aim of evaluating how patients perceive their illness during the β-hCG follow-up period after GTD diagnosis. We first of all wanted to evaluate if there were significant differences in mental illness representations between benign trophoblastic tumor patients (HM) and patients diagnosed with malignant forms of GTD (GTN). Secondly, we aimed to assess if demographic and medical variables (such as time passed since diagnosis) correlate significantly with the scores patients obtained on specific illness perceptions subscales. The overall aim of the study was to explore mental illness representations in women diagnosed with these rare trophoblastic tumors in order to provide support to these women in an outpatient clinic setting.

## Materials and Methods

### Sample Selection and Recruitment

Patients treated for GTD at a Hospital in Northern Italy were invited to take part in the research project. Women were recruited in the outpatient Gestational Trophoblastic Hospital Clinic; no patient declined to take part in the study. Eligible women had to be Italian-speaking, with at least an elementary school certificate and agreed voluntarily to participate in the research. Women who were taking psychiatric medication or who had previously had a chronic illness were excluded from the study. We also asked women if they were currently seeing or had seen a psychologist or psychiatrist in the past. If we deemed that women had an ongoing psychiatric morbidity, they were excluded from the study. Based on these criteria 2 women were excluded from the study and 31 women took part (N = 31); 8 women were diagnosed with GTN and the remaining 23 patients had pre-malignant forms of GTD (partial or complete hydatidiform mole). The age range of our sample was from 16 to 56 years (mean age = 35.97; SD = 9.745). The mean time elapsed from the moment of diagnosis to questionnaire completion was 4.65 months (range = 1–25; SD = 4.652).

The study was approved by the Medical Ethical Committee of the San Raffaele Hospital on May 6th, 2010. A written informed consent was obtained by all the participants at the time of questionnaire completion. With regards to the consent for minors, at questionnaire completion, we obtained verbal consent from the parents of the minors and the parents were present during the entire testing phase. The minors gave their verbal consent too. We have not used the data from the minors until they turned 18. At that very moment, they signed their consent forms autonomously and we have now decided to incorporate their data in our study. The Medical Ethical Committee approved this consent process.

### Measures

Demographic and clinical information were collected through the use of a self-report questionnaire which included date of birth, level of education, relationship status, parity, date of diagnosis, type of diagnosis and nature of therapy used to treat the disease.

To assess patients’ illness perceptions the *Illness Perception Questionnaire-Revised* (IPQ-R) was administered [23]. The IPQ-R is divided into three sections. The *Identity* scale (14 items) measures, through a yes/no response format, the number of symptoms patients have experienced and whether these symptoms are perceived to be related to their illness. The second section evaluates patients’ opinions regarding their illness using a 5-point Likert scale (from 1 “strongly disagree” to 5 “strongly agree”). This scale is composed of 38 items which investigate how long subjects think their condition will last (*Timeline acute-chronic*); whether symptoms are expected to be sustained or cyclical (*Timeline cyclical*); the perceived *Consequences* of the illness; how much control patients feel they have over the illness (*Personal control*); their beliefs about the treatment’s efficacy (*Treatment control*); whether they have a coherent understanding of the condition (*Illness coherence*) and the emotional responses generated by the illness (*Emotional representations*). Lastly, the *causes* subscale examines the explanations patients use to represent their illness. This dimension, which is composed of 18 items rated on the same Likert-type scale, consists of three principal factors: stress or worry, hereditary causes and chance or bad luck [12, 24]. The IPQ-R has shown good internal reliability and structural validity in previous research [23]. In this study we use the Italian version of the IPQ-R [25].

### Statistical analysis

Statistical analyses were conducted using SPSS software, version 20.0 [26].

Patients were grouped on the basis of the type of diagnosis: hydatidiform mole (HM) or gestational trophoblastic neoplasia (GTN). A Mann-Whitney test was performed in order to assess differences between the two subgroups. Correlations between demographic variables and illness perception dimensions were evaluated by virtue of the Spearman’s correlation coefficient ρ_s_. The level of significance was set at p < .05.

## Results and Discussion

### Sample Characteristics

Sample characteristics were investigated separately for HM and GTN patients in order to evaluate possible differences between the two groups of participants (Table 1). With regards to the HM group of participants, the majority of women declared that when they completed the questionnaires they were in a stable relationship (95.7%; N = 22). Specifically, the majority of women were married (82.6; N = 19), 8.7% (N = 2) were single, and 8.7% (N = 2) were living with their partner. About 41.9% (N = 13) of the women had had a child prior to the GTD diagnosis, whereas for 43.5% (N = 10) this was their first pregnancy at the time of diagnosis. With respect to the GTN group of participants, the majority of women declared that when they completed the questionnaires they were in a stable relationship (87.5%; N = 7). Specifically, the majority of women were married (75.0%; N = 6), 12.5% (N = 1) were single, and 12.5% (N = 1) were living with their partner. About 62.5% (N = 5) of the women had had a child prior to the GTD diagnosis, whereas for 37.5% (N = 3) this was their first pregnancy at the time of diagnosis. No patient declared to have had a child after GTD as all the women in our sample were in their β-hCG follow-up period when the questionnaires were administered. All patients with GTN were treated with chemotherapy, while women with HM diagnosis were undertaking only gonadotropin (β-hCG) follow-up except for two women who underwent total hysterectomy.

**Table 1:** Patient and Illness-related characteristics separated on the basis of the type of diagnosis (hydatidiform mole and gestational trophoblastic neoplasia).

	HM		GTN		
	N	%	N	%	χ^2^
*Marital status*					.22 (.90)
**Married**	19	82.6	6	75.0	
**Cohabiting**	2	8.7	1	12.5	
**Single**	2	8.7	1	12.5	
*Profession*					1.7 (.63)
**Employed**	17	73.9	6	75.0	
**Freelancer**	3	13.0	0	0.0	
**Unemployed**	1	4.3	1	12.5	
**Student**	2	8.7	1	12.5	
*Presence of children*					.09 (.77)
**Yes**	10	43.5	3	37.5	
**No**	13	41.9	5	62.5	

HM = hydatidiform mole group (N = 23); GTN = gestational trophoblastic neoplasia group (N = 8); p-values are in brackets.

No significant differences were found for any of the demographic variables between the HM and the GTN group of participants.

### Illness perception results

Means, standard deviations and IPQ scale ranges are reported in Table 2. On the Identity scale the mean score obtained by patients was 2.81 (SD = 2.587), suggesting that women tend to perceive GTD as a condition characterized by a relatively restrained symptomatology. Specifically, the symptoms that participants most frequently associated to GTD were: fatigue (reported by 51.6% of patients), followed by nausea (48.4% of patients) and loss of strength (32.3% of patients).

**Table 2:** Means, standard deviations, ranges, minimum and maximum scores of the IPQ-R Identity and Illness opinions subscales.

IPQ-R subscales	Range	M (SD)	Min.	Max.
**Identity**	0–14	2.81 (2.587)	0	9
**Timeline acute/chronic**	6–30	12.68 (3.439)	6	21
**Timeline cyclical**	4–20	9.13 (3.344)	4	16
**Consequences**	6–30	16.23 (3.575)	10	26
**Personal control**	6–30	16.16 (4.960)	6	30
**Treatment control**	5–25	19.77 (2.883)	13	25
**Illness coherence**	5–25	17.65 (3.342)	11	25
**Emotional representations**	6–30	19.84 (4.495)	10	30

Regarding GTD illness opinions, the highest mean scores were observed on the Emotional representations (mean = 19.84; SD = 4.495) and Treatment control (mean = 19.77, SD = 2.883) subscales. Elevated scores in the former dimension are indicative of a predominance of negative emotions developed in response to illness whereas high scores in the latter dimension reflect confidence in treatment efficacy and in the possibility of exerting a positive influence on the evolution of the illness. Scores obtained on the Illness coherence subscale (mean = 17.65; SD = 3.342) suggest relatively good coherence of the illness representations and a good level of understanding of the medical situation (Table 2).

With respect to the causes of GTD, patients’ opinions are reported in Table 3. When considering single items within the psychological attributions subscale, the highest mean score was observed in relation to the “stress/worry” causal factor (mean = 2.68; SD = 1.423). Within the Risk factors subscale “ageing” presented the highest score (mean = 2.42; SD = 1.148). Regarding the Immunity subscale the highest mean score was reported on the “pollution in the environment” item (mean = 5.29; SD = 1.092). Within the Accident/chance subscale the factor with the highest score was “chance/bad luck” (mean = 3.81; SD = 1.276). Considering each possible cause singularly, the highest mean scores were observed in this order: chance/bad luck, followed by stress/worry and then pollution in the environment.

**Table 3:** Means, standard deviations, ranges, minimum and maximum scores of the IPQ-R “causes” subscale.

IPQ-R causes subscales	Range	M (SD)	Min.	Max.
**Psychological attributions**	6–30	11.58 (4.738)	6	24
**Risk factors**	7–35	13.26 (3.838)	7	23
**Immunity**	3–15	6.61 (2.404)	3	13
**Accident/chance**	2–10	5.29 (1.488)	2	8

Table 4 shows that a significant difference emerged between patients affected by hydatidiform mole (HM) and those with gestational trophoblastic neoplasia (GTN) on the Identity subscale of the IPQ-R (U = 43.50, p < .03) whereby women affected with GTN report a significantly higher score (Mdn = 5.00) when compared to HM patients (Mdn = 1.00). No other significant differences were observed by these two diagnostic subsamples.

**Table 4:** Diagnostic differences (HM versus GTN) on the IPQ-R subscales.

IPQ-R subscales	HM	GTN	
	(N = 23)	(N = 8)	
	Mdn	Mdn	Mann-Whitney U
**Identity**	1.00	5.00	43.50*
**Timeline acute/chronic**	13.00	14.00	63.00
**Timeline cyclical**	10.00	9.00	85.50
**Consequences**	15.00	18.00	52.00
**Personal control**	15.00	16.00	86.00
**Treatment control**	20.00	20.00	86.00
**Illness coherence**	17.00	17.50	80.50
**Emotional representations**	20.00	23.50	53.00
**Psychological attributions**	12.00	9.50	71.50
**Risk factors**	13.00	12.50	81.50
**Immunity**	6.00	6.50	85.00
**Accident/chance**	6.00	6.00	89.50

*p = .03

HM = hydatidiform mole; GTN = gestational trophoblastic neoplasia.

Lastly correlation analyses were conducted to evaluate the relationship between demographic variables and IPQ-R subscales. The results reported in Table 5, show the presence of a significant positive correlation between “time since diagnosis” and the Treatment control subscale on the IPQ-R (ρ = .426, p = .02).

**Table 5:** IPQ-R subscale correlations with patient age and time since diagnosis.

IPQ-R subscales	Age	Time since diagnosis
	Spearman ρ	Spearman ρ
**Identity**	-.222	.333
**Timeline acute/chronic**	.241	-.204
**Timeline cyclical**	-.066	-.193
**Consequences**	.144	.029
**Personal control**	.072	.294
**Treatment control**	-.151	.426*
**Illness coherence**	-.359	-.130
**Emotional representations**	.101	-.069
**Psychological attributions**	.094	-.123
**Risk factors**	-.110	.003
**Immunity**	.023	-.151
**Accident/chance**	-.276	.006

* p = .02

## Discussion

At present Hydatidiform Mole and Gestational Trophoblastic Neoplasia are both highly curable diseases [27]. Despite the fact that a full recovery is generally expected, women diagnosed with GTD have to confront the loss of a pregnancy, a potentially life-threatening diagnosis, surgical and/or chemotherapy treatment and delays in future pregnancies [28]. Currently, the psychological impact of this condition for both the patient and her partner has been studied; focus has been specifically on the psychopathological consequences of the disease [8–10, 28–30], on patient quality of life [10, 30–32] and on fertility-related stress [8, 9, 31, 33]. One of the largest studies in GTD to date highlighted that psychological morbidity in GTD patients exceeds community norms, especially when it comes to depression and anxiety [34].

The present study has the aim of expanding on previous research in this area to explore the mental representations of illness that patients with GTD present. In particular, specific dimensions of illness perception were analyzed and significant differences were explored with regards to the type of GTD diagnosis. Other medical variables (such as time since diagnosis and type of diagnosis) and demographic characteristics (age) were also examined and controlled for.

The statistical analyses show that patients within our sample reported a relatively weak illness Identity. When comparing our results with those of studies that previously investigated illness perception in other female tumors (such as breast cancer) [35, 36], the mean number of symptoms that the patients in our sample associated to their disease was in fact rather low. In detail, fatigue was the most reported symptom where 51.6% of our sample described this clinical manifestation, followed by nausea (48.4%) and loss of strength (32.3%). Interestingly, illness Identity is the only dimension on the IPQ-R that showed a significant difference between women diagnosed with premalignant hydatidiform mole and those diagnosed with malignant GTN (p < .05). This result seems to reflect a real difference between the two diagnoses with regards to the clinical presentation. Molar pregnancies (HM) are usually diagnosed during the first trimester of pregnancy and present as pregnancy failure. The diagnosis is histological and made after uterine evacuation. Around 10–15% of patients with a previous diagnosis of HM finally develop a GTN, this diagnosis is made via β-hCG testing. Many patients with either complete or partial HM are asymptomatic [37, 38], however, among symptomatic patients, the most common presenting symptom is abnormal bleeding [39]. Other symptoms, which might correlate with a higher risk of developing GTN, are an enlarged uterus, irregular bleeding and persistent bilateral ovarian enlargement. GTN might also be diagnosed after a term pregnancy or a non-molar miscarriage. In more severe cases, bleeding as a result of uterine perforation or metastatic lesions may result in abdominal pain, hemoptysis or melena. Furthermore, patients with GTN and central nervous system metastases often exhibit evidence of increased intracranial pressure from intra-cerebral hemorrhage, which may lead to headaches, seizures or hemiplegia. Patients may also present with pulmonary symptoms, such as dyspnea cough and chest pain, caused by extensive lung metastases [3]. Although these specific symptoms are not directly measured by the IPQ-R questionnaire, we hypothesize that the more severe clinical presentation of GTN, together with their more invasive treatment, may affect patients’ illness Identity representations, which are more serious in GTN when compared to HM.

With regards to patients opinions surrounding their illness, the highest mean scores were found on the Emotional representations and the Treatment control subscales of the questionnaire. Regarding Emotional representations, high scores indicate a response to illness characterized prevalently by negative emotions, reflecting intense emotional reactions that a disease, such as cancer can invoke. Fear and anxiety together with symptoms of abandonment and anger, invoked from the sense of vulnerability and loss of control of one’s life, represent the most frequent psychological reactions when a person discovers a potentially lethal disease and its consequent treatment [40]. A GTD diagnosis could amount to a psychological shift, overwhelming and changing a patient’s life and future plans, forcing the patient to redefine herself with regards to existential goals [41]. The experience that GTD entails, for example distress tied to treatments and follow-up, could account for the high score on the Emotional representations subscale. A previous study supports this finding, Petersen and colleagues [10] found that intense emotional reactions were present in their sample of GTD patients. These authors specify that shock, feelings of sadness or depression (induced primarily by the diagnosis and the need to postpone a future pregnancy), feelings of uncertainty (regarding the causes of illness), anxiety (tied to specific characteristics of the disease and its possible consequences), worries about future fertility and the possibility of a relapse represent the most common emotions invoked by GTD in female patients. Regarding Treatment control, patients reported a high mean score, demonstrating confidence and a certain degree of control over the treatment. This result is in line with previous research [33] that analyzed perceived causes and treatment in the contest of GTD. The authors highlight how all women with GTN were aware of the need for chemotherapy, specifying that most women considered the treatment effective and knew which chemotherapeutic agent was being administered.

Correlational analyses show that the Treatment control subscale is positively correlated with “time since diagnosis”. This is in contrast with previous studies that suggest that illness perception is relatively stable over time [42–44]; this research is characterized by the fact that participants did not have the support of a multidisciplinary team, including psychological support. The result of our study can be interpreted in light of the fact that a specific service and psychological support are offered to the patients by the healthcare staff at this particular hospital in Italy. The presence of a multidisciplinary team (made up of gynecologists, nurses and psychologists) which supports patients from the moment a diagnosis is communicated all the way to the end of follow-up could promote a more supportive climate that welcomes insecurities, incomprehensions and emotions tied to GTD. The regular contact with medical and psychological staff could contribute to a more realistic illness perception as well as willingness to have confidence in treatment and future fertility, and also acquisition and reinforcement of self-efficacy. This could, in time, help develop a higher confidence in treatment effectiveness and a perceived control over treatment. This hypothesis is supported by Paschali and colleagues [45] who highlight the presence of a significant correlation between the amount of information transmitted by the treating team (concerning diagnosis and treatment) and the tendency of patients to develop a more accurate and positive mental representation of illness. In particular, the more information patients received, the less the impact of the cancer diagnosis and the more control they felt over the disease.

With regards to the perception of the causes of GTD, the highest mean score was associated to “chance/bad luck”. This is consistent with previous studies [33, 46] that report that the majority of patients have the tendency to attribute their condition to chance. This can be due to the fact that during their first gynecological visit patients are informed that the GTD pathogenesis is mainly linked to a very rare chromosomal alteration that occurs during the fertilization event between an ovum and a spermatozoon. One could hypothesize that the rarity of this abnormal fertilization event may lead patients to re-elaborate this causal explanation in terms of chance or bad luck. Moreover, within the IPQ causes subscale there is no item that refers to genetics or chromosomal alterations specifically, thus patients could identify “chance/bad luck” as the most suitable causal explanation of their condition. From a psychological point of view one must consider that the discovery of the disease in a specific moment of one’s life (i.e., maternity) together with the feelings of frustration and impotence (tied to the interruption of a pregnancy and its consequences on future plans) could induce patients to search for the cause of their disease in an external, incontrollable and predetermined agent, such as fate. Moreover, it is important to point out that, while the women in our sample reported an elevated level of education, a percentage of women in previous studies [33, 46] who had lower levels of education and lower socio-economic status presented the same result. The fact that in all samples (including our own) women seemed to attribute the cause of GTD to chance or bad luck could suggest that this result is common to most patients with a GTD diagnosis, irrelevant of education or cultural level.

Results of the present study must be read in the light of certain limitations. Among these, the most salient is the small sample size. Given the rarity of gestational trophoblastic tumors and the fact that we did not use a tumor registry but rather we preferred to hand out our questionnaires in person to our patients, we feel that the study is still very much pertinent and important in research. We also feel that a lack of a control group could make it difficult to interpret the data. In the future we would like to add a control group of patients who have similar conditions for example, diseases characterized by a very high cure rate but also a long follow-up with risk of developing into a malignant cancer. We do, however, feel that this study is a good starting point to measure illness perception in GTD. The generalizability of our results is also limited due to the fact that our sample was mostly made up of Italian women with a medium-high education level. A limitation that came to light as we conducted the study was that there was no causal item in IPQ-R relating to a chromosomal alteration or genetic abnormality and thus women could not choose the true cause of GTD in their options on the questionnaire when identifying the factors responsible for the disease. Perhaps in the future the causal subscale could be modified to accommodate such a causal explanation. Lastly, the cross-sectional design of our study could be re-evaluated in the future; a measurement at only one point in time is limiting and it would be interesting to analyze possible changes in illness perception over time and try to identify causal relationships between the variables that were measured in this study.

## Conclusions and Future Research

The present research is the first study to systematically investigate illness perception in GTD patients. Although in our sample women in general did not report an elevated number of symptoms, GTN patients presented a significantly stronger illness Identity compared to HM. This study highlights how GTD is accompanied by significant emotional reactions for women who are diagnosed with this group of disorders, supporting results from previous research [11, 41, 47]. Despite the significant emotional responses generated by GTD, regular support and communication offered by healthcare staff in our hospital seem to promote a Treatment control in patients that develops gradually over time. Therefore, from a clinical point of view the results of this study highlight the need to incentivize multidisciplinary support programs that aim to promote overall well-being.

It is nevertheless necessary to replicate this study, especially in a longitudinal manner so as to observe changes in illness perception over time. In the future it is also important to examine illness perception in larger samples of patients so that the participants could be divided into subsamples of specific GTD diagnoses. Furthermore, the presence of a control group could help interpret the results in a more accurate manner. Lastly, in light of the evidence presented in the literature, which suggests that mental representations of illness can play a role in modulating psychological adaptation of patients with an organic disease [13, 48–50], it would be interesting to evaluate the specific impact that illness perception could have on levels of anxiety, depression and infertility-related stress in women with GTD.

Ultimately, we suggest that the minimum standard of care should involve psycho-educational interventions related to the disease, to the treatment and its side effects, and reassurance related to generally favorable prognoses as well as fertility preservation after a cure from GTD has been obtained. This would help to allay fears, enhance compliance, quality of life and control over the disease and treatment.
